# Evidence-based Medication knowledge Brokers in Residential Aged CarE (EMBRACE): protocol for a helix-counterbalanced randomised controlled trial

**DOI:** 10.1186/s13012-024-01353-z

**Published:** 2024-03-04

**Authors:** J. Simon Bell, Adam La Caze, Michelle Steeper, Terry P. Haines, Sarah N. Hilmer, Lakkhina Troeung, Lyntara Quirke, Jacqueline Wesson, Constance Dimity Pond, Laurie Buys, Nazanin Ghahreman-Falconer, Michael T. Lawless, Shakti Shrestha, Angelita Martini, Nancy Ochieng, Francesca Glamorgan, Carmela Lagasca, Rebecca Walton, Dayna Cenin, Alison Kitson, Monica Jung, Alexandra Bennett, Amanda J. Cross

**Affiliations:** 1 Centre for Medicine Use and Safety, Faculty of Pharmacy and Pharmaceutical Sciences, Monash University, Parkville Campus, Parkville, VIC 3052 Australia; 2 School of Pharmacy, The University of Queensland, Dutton Park, QLD Australia; 3 Faculty of Medicine Nursing and Health Sciences, School of Primary and Allied Health Care, Monash University, Clayton, VIC Australia; 4 National Centre for Healthy Ageing, Frankston, VIC Australia; 5 Kolling Institute, Faculty of Medicine and Health, The University of Sydney and Northern Sydney Local Health District, St Leonards, NSW Australia; 6 New South Wales Therapeutic Advisory Group, Sydney, NSW Australia; 7 Brightwater Research Centre, Brightwater Care Group, Inglewood, WA Australia; 8 Dementia Australia, Canberra, Australia; 9 Ageing and Health Research Unit, Discipline of Occupational Therapy, Faculty of Medicine and Health, Sydney School of Health Sciences, University of Sydney, Camperdown, NSW Australia; 10 Wicking Dementia Research and Teaching Centre, University of Tasmania, Hobart, TAS Australia; 11 Faculty of Health Sciences, Australian Catholic University, Brisbane, QLD Australia; 12 Pharmacy Department, Princess Alexandra Hospital, Woolloongabba, QLD Australia; 13 Caring Futures Institute, College of Nursing and Health Sciences, Flinders University, Bedford Park, Adelaide, South Australia Australia; 14 Lifeview Corporate Lifeview Pty Ltd., Carnegie, VIC Australia; 15 Montefiore, Randwick, NSW Australia; 16 Anglicare Southern Queensland, Fortitude Valley, QLD Australia

**Keywords:** Long-term care, Residential facilities, Randomised controlled trial, Implementation science, Practice guidelines as topic, Implementation strategies, Inappropriate prescribing, Dementia, Evidence-based practice, Psychotropic drugs

## Abstract

**Introduction:**

Clinical practice guidelines recommend against the routine use of psychotropic medications in residential aged care facilities (RACFs). Knowledge brokers are individuals or groups who facilitate the transfer of knowledge into practice. The objective of this trial is to evaluate the effectiveness and cost-effectiveness of using knowledge brokers to translate Australia’s new Clinical Practice Guidelines for the Appropriate Use of Psychotropic Medications in People Living with Dementia and in Residential Aged Care.

**Methods and analysis:**

The Evidence-based Medication knowledge Brokers in Residential Aged CarE (EMBRACE) trial is a helix-counterbalanced randomised controlled trial. The 12-month trial will be conducted in up to 19 RACFs operated by four Australian aged care provider organisations in Victoria, New South Wales, Western Australia and Queensland. RACFs will be randomised to receive three levels of implementation strategies (knowledge broker service, pharmacist-led quality use of medications education activities and distribution of the Guidelines and supporting materials) across three medication contexts (antipsychotics, benzodiazepines and antidepressants). Implementation strategies will be delivered by an embedded on-site aged care pharmacist working at a system level across each participating RACF. All RACFs will receive all implementation strategies simultaneously but for different medication contexts. The primary outcome will be a composite dichotomous measure of 6-month RACF-level concordance with Guideline recommendations and good practice statements among people using antipsychotics, benzodiazepines and antidepressants for changed behaviours. Secondary outcomes will include proportion of residents with Guideline concordant use of antipsychotics, benzodiazepines and antidepressants measured at the RACF-level and proportion of residents with psychotropic medication use, hospitalisation, falls, falls with injury, polypharmacy, quality of life, activities of daily living, medication incidents and behavioural incidents measured at the RACF-level.

**Discussion:**

The EMBRACE trial investigates a novel guideline implementation strategy to improve the safe and effective use of psychotropic medications in RACFs. We anticipate that the findings will provide new information on the potential role of knowledge brokers for successful and cost-effective guideline implementation.

**Trial registration:**

Australian New Zealand Clinical Trials Registry (ANZCTR): ACTRN12623001141639. Registered 6 November 2023 — retrospectively registered, https://www.anzctr.org.au/TrialSearch.aspx.

**Supplementary Information:**

The online version contains supplementary material available at 10.1186/s13012-024-01353-z.

Contributions to the literature

- This trial is one of the first to directly compare different guideline implementation strategies to improve concordance with clinical practice guidelines in residential aged care facilities.
- This trial will utilise a helix-counterbalanced study design to simultaneously deliver and evaluate multiple strategies to improve appropriate use of psychotropic medications across residential aged care facilities.
- This trial will evaluate a novel role for pharmacists as system-level knowledge brokers in residential aged care facilities.
- If successful, the guideline implementation strategies may be applicable to other therapeutic areas and contexts to improve the quality use of medications for residents living in residential aged care facilities.

## Background

Persistent high rates of psychotropic medication use in residential aged care facilities (RACFs) remain a concern for residents, caregivers and other stakeholders. This is despite a lack of evidence supporting benefits of psychotropic medication but well-documented risks of harm. More than 60% of Australian RACF residents use regular psychotropic medications, with 41% prescribed antidepressants, 22% antipsychotics and 22% benzodiazepines on a regular basis [1]. Psychotropic medication prevalence is also highly variable, with a study across 27 RACFs in the state of Victoria reporting antipsychotic prevalence ranged from 0 to 95% (mean 32%) and antidepressant prevalence ranged from 10 to 89% (mean 48%) [2].

The American Psychiatric Association [3], European Academy of Neurology [4] and Australian Clinical Practice Guidelines and Principles of Care for People with Dementia [5] recommend avoiding antipsychotics in people living with dementia. Evidence suggests antipsychotics are associated with increased risk of cardiovascular and cerebrovascular events, falls and death [6, 7]. Benzodiazepines are associated with minimal and short-term improvements in sleep disturbances but also with an increased risk of hip fracture [8] and falls [9]. Antidepressants have uncertain benefits in people living with dementia and have been associated with adverse events including dry mouth, dizziness and falls [10].

In October 2019, Australia’s Royal Commission into Aged Care Quality and Safety identified overreliance on psychotropic medications for chemical restraint as one of three areas for immediate action [11]. The National Health and Medical Research Council (NHMRC) Dementia Centre for Research Collaboration subsequently commissioned new Australian Clinical Practice Guidelines for the Appropriate Use of Psychotropic Medications in People Living with Dementia and in Residential Aged Care (the Guidelines) [12]. The Guidelines were developed by a multidisciplinary 18-member Guideline Development Group, supported by a Stakeholder Advisory Committee comprising government agencies, professional groups, aged care provider organisations and quality improvement organisations. The Guidelines were developed using an integrated knowledge translation process in collaboration with five aged care provider organisations in Queensland, South Australia, Western Australia and New South Wales [13, 14]. Ongoing focus groups with these organisations suggested that innovative guideline implementation strategies would be needed to fully implement guideline recommendations and good practice statements.

Poor implementation of guideline recommendations is recognised as a barrier to reducing medication-related harm [15]. There is emerging interest in engaging knowledge brokers to support guideline implementation [16]. Knowledge brokers are individuals or groups that help to move knowledge from those who create the knowledge (e.g. guideline developers) to those that use the knowledge (e.g. health care professionals) [16]. Knowledge brokers act as knowledge managers, linking agents and capacity builders [16]. The role of a knowledge broker is consistent with the Australian Government’s recent announcement of AUD $345.7 million over 4 years for on-site embedded pharmacists in all Government-funded RACFs to improve medication safety [17]. Quality Use of Medicines (QUM) and Medicines Safety is also Australia’s newest National Health Priority Area [18]. In recognition that evidence-based recommendations alone are not sufficient to ensure guideline-concordant appropriate medication use, we developed a series of data-driven knowledge translation strategies for implementing the new Guidelines.

The objective of this trial is to evaluate the effectiveness and cost-effectiveness of using knowledge brokers to translate Australia’s new Clinical Practice Guidelines for the Appropriate Use of Psychotropic Medications in People Living with Dementia and in Residential Aged Care. Guideline implementation using knowledge brokers will be compared to guideline implementation through the following: (1) distribution of the Guidelines and supporting materials and (2) pharmacist-led QUM education activities.

## Methods

### Design

**Fig. 1:**
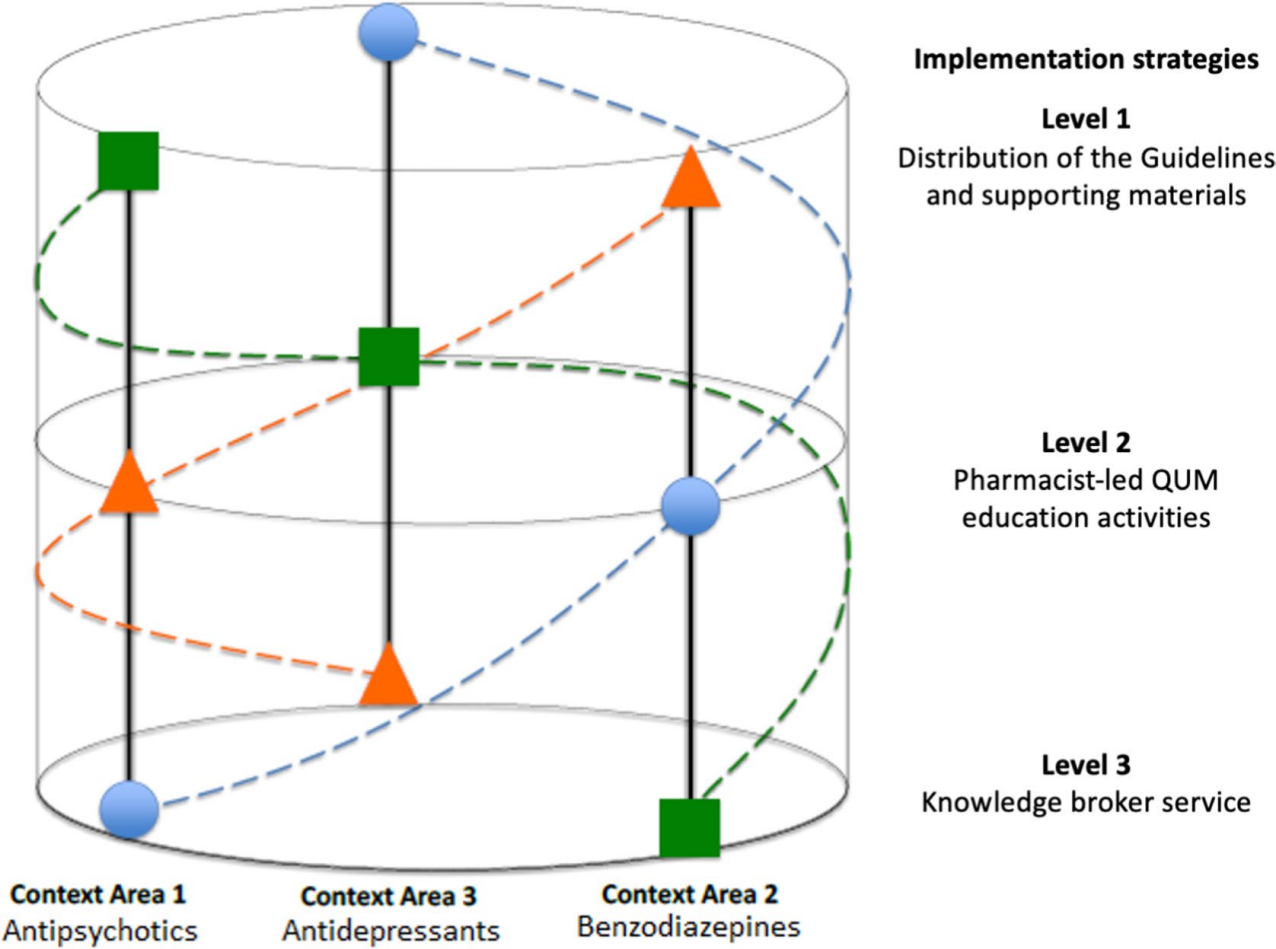
Helix counterbalanced design illustrating context area-by-intervention level combinations for three groups of residential aged care facilities (square, circle and triangle). This Figure was adapted with permission from Sarkies et al. Implementation Science 2019;14:45. Abbreviations: QUM, quality use of medicine

The *E*vidence-based *M*edication knowledge *B*rokers in *R*esidential *A*ged *C*ar*E* (EMBRACE) trial is a 12-month helix-counterbalanced randomised controlled trial [19]. The trial will examine three different guideline implementation strategies (Fig. 1). These strategies will be evaluated across the three psychotropic medication contexts included in the new Guidelines (antipsychotics, benzodiazepines and antidepressants) [12]. All participating RACFs will receive all implementation strategies but for different medication contexts (Table 1, Fig. 1). This trial will involve RACF-level interventions that will not involve the investigators collecting resident-level identifiable data.

**Table 1:** Intervention combinations delivered for each RACF group

Group	Levels	Knowledge translation strategy to medication context area
Group A RACF	**Level 1**	Distribution of hard copy and electronic guideline on *antidepressants*
	**Level 2**	Distribution of the Guidelines and QUM education sessions on *benzodiazepines*
	**Level 3**	Knowledge broker led local action plans, QUM education sessions and distribution of the Guidelines on *antipsychotics*
Group B RACF	**Level 1**	Distribution of hard copy and electronic guideline on *antipsychotics*
	**Level 2**	Distribution of the Guidelines and QUM education sessions on *antidepressants*
	**Level 3**	Knowledge broker led local action plans, QUM education session and distribution of the Guidelines on *benzodiazepines*
Group C RACF	**Level 1**	Distribution of hard copy and electronic guideline on *benzodiazepines*
	**Level 2**	Distribution of the Guidelines and QUM education sessions on *antipsychotics*
	**Level 3**	Knowledge broker led local action plans, QUM education session and distribution of the Guidelines on *antidepressants*

Abbreviations: *RACF*, residential aged care facility; *QUM*, quality use of medicine

In advance of the trial, the Project Management Team met with each participating aged care provider organisation’s leadership team to discuss the trial design and delivery. Design of this trial was also informed by a series of foundational activities including the following:

- An integrated knowledge translation process involving an ongoing series of focus groups with aged care provider organisations to develop the new Clinical Practice Guidelines for the Appropriate Use of Psychotropic Medications in People Living with Dementia and in Residential Aged Care [12, 14].
- In-depth semi-structured interviews and focus groups (*n* = 33) involving RACF managers, prescribers, pharmacists, nurses, aged care workers, residents, carers and other key stakeholders at participating aged care provider organisations to assess each aged care provider organisation’s existing quality improvement processes [20].
- Fifteen semi-structured interviews with early adopters of Australia’s embedded on-site aged care pharmacist model to understand potential synergies with our knowledge broker role and to inform the development of knowledge broker training [21].
- Systematic review of the published literature on the roles and effectiveness of knowledge brokers in implementing clinical practice guidelines [16].

### Setting

The trial will be conducted in up to 19 RACFs operated by four aged care provider organisations in the states of Victoria, New South Wales, Western Australia and Queensland. The four aged care provider organisations are non-government, independent and not-for-profit organisations. Australian RACFs provide supported accommodation for people with care needs that can no longer be met in their own homes and are synonymous with long-term care facilities and nursing homes in other countries [22, 23]. Approximately, two thirds (65%) of residents in Australian RACFs are women, and 58% of residents are aged 85 years and over [24]. In Australian RACFs, medications are mostly prescribed by visiting medical practitioners, dispensed by off-site community pharmacies and administered by registered nurses or credentialled aged care workers [25]. Each aged care provider organisation’s leadership team will be responsible for identifying the specific RACFs operated by that organisation to take part in the trial. The trial protocol conforms to the Standard Protocol Items: Recommendations for Interventional Trials (SPIRIT) guidelines [26, 27] (Additional file 1). The trial has been registered with the Australian and New Zealand Clinical Trials Registry (ANZCTR) (registration no.: ACTRN12623001141639, date registered: 06/11/2023). The application for trial registration was submitted on the 20th of September 2023 (as shown on the ANZCTR trial registration site) prior to randomisation of RACFs, but the trial was not formally registered until 6th November 2023. There were no changes to the trial protocol between the submission date (20th of September 2023) and the registration date (6 November 2023).

### Governance and consultation

The trial will be overseen by the Project Management Team comprising the lead investigators and project manager. During the trial, the Project Management Team will meet with the aged care provider organisation representatives and knowledge brokers monthly or more frequently if required to monitor the data collection and conduct of the trial and to identify any potential adverse events. Meetings will mostly be conducted via video-teleconferencing (e.g. Zoom), with face-to-face meetings organised if needed.

The trial will be supported by a stakeholder group comprising government agencies (Aged Care Quality and Safety Commission, Australian Commission on Safety and Quality in Health Care), professional groups (Royal Australian and New Zealand College of Psychiatrists, Australian and New Zealand Society of Geriatric Medicine, Australian College of Nursing, Pharmaceutical Society of Australia, Pharmacy Guild of Australia), and an advocacy body (Dementia Australia). The stakeholder group will meet annually for the duration of the trial including at implementation, evaluation and dissemination stages. A Data Safety Monitoring Board will meet as and when required during the trial. It will involve an independent group of healthcare professionals with relevant clinical expertise (e.g. general practitioner, geriatrician, nurse, pharmacist).

### Randomisation and blinding

This trial will involve RACF-level interventions, and individual residents will not be recruited to participate. The unit of randomisation will be RACFs. Once potential availability of specific RACFs is determined by each aged care provider organisation, the RACF manager or an authorised representative of the aged care provider organisation will be provided with a written explanatory statement, and informed consent to participate will be obtained.

RACFs will be matched into blocks of three based on the aged care provider organisation and geographical location. One RACF within each block of three will be randomised to groups A, B, or C and receive three levels of intervention for a combination of implementation strategies and medication contexts (Table 1). Randomisation will be performed by an independent epidemiologist using a computerised random number generator within SAS (SAS Institute, Cary, NC, USA).

Following randomisation, each aged care provider organisation leadership team and their knowledge brokers will be notified of group allocations for each participating RACF within their organisation. Due to the nature of the trial, it will not be possible to blind the leadership team or knowledge brokers. Partial disclosure will be implemented for RACF nurses, aged care workers and visiting pharmacist and medical practitioners at each RACF. These clinicians and staff will be aware of the trial objectives and implementation strategies but will be blinded to the RACF group allocation. The availability of the knowledge broker service will be communicated via letters, posters and notice boards. Residents and their families will be made aware of the trial through distribution of notices and posters at each RACF. The primary outcome will be analysed by a blinded statistician or epidemiologist. The Project Management Team will not be blinded to the group allocations.

### Knowledge brokers

This trial will evaluate a complex intervention involving on-site embedded pharmacists working as knowledge brokers at each aged care provider organisation. Each aged care provider organisation will recruit and employ or contract their own knowledge brokers. The Project Management Team will engage with each aged care provider organisation to help identify suitable knowledge brokers if required. Knowledge brokers will be identified through an expression of interest process via direct approaches to the aged care provider’s existing pharmacy service provider, direct approach to known professional contacts of the Project Management Team or aged care provider organisation or via local advertisements.

Pharmacists will be eligible to be a knowledge broker if they:

- Hold general pharmacist registration with the Australian Health Practitioner Regulation Agency.
- Are willing and available to commit to undertake the role for 12 months, including completing training and specific tasks of the trial.
- Are willing and able to complete all relevant aged care provider organisation induction and occupation, health and safety training requirements and be contracted or employed to work with each aged care provider.

It will be desirable for the knowledge brokers to have prior aged care experience and accreditation to complete medication management reviews (MMRs), but this will not be a requirement [28]. Potential knowledge brokers will be provided with an explanatory statement, and written informed consent will be sought from the knowledge brokers. Knowledge brokers will be trained to support delivery of the implementation strategies by undergoing a training programme over a 4–5-week period prior to trial commencement. The training program consists of the following:

- 3 × 1.5-h online workshops on knowledge translation delivered by implementation science experts. The workshops include one self-paced and two interactive modules that cover evidence implementation, stakeholder engagement, driving meaningful change and the integrated-Promoting Action on Research Implementation in Health Services (i-PARIHS) framework. These modules have been developed for the purpose of the trial and were informed by the foundational activities described earlier.
- A 1.5-h online webinar on the EMBRACE trial design and the intervention and guidance on how to deliver the implementation strategies delivered by the Project Management Team.
- A 1.5-h webinar on the Guidelines delivered by the Project Management Team [12]
- Residential Aged Care Pharmacist Foundation Training Program developed and delivered by the Pharmaceutical Society of Australia [29]. This 9-h programme involves four residential aged care online modules, nine video lectures on aged care clinical topics, three optional modules dedicated to QUM and two optional modules on health literacy and digital literacy.

### Intervention

All three levels of implementation strategies will be delivered simultaneously throughout the 12-month intervention period at the RACF-level. Each level of implementation strategy includes the activities of the previous level (Table 1). Education resources will be provided to support knowledge brokers to deliver level 1 and level 2 implementation strategies. Level 3 implementation strategies will be customised to each RACF based on ongoing local needs assessments conducted by the knowledge broker.

#### Level 1: Distribution of the Guidelines and supporting materials

The level 1 strategy will involve passive distribution of the Guidelines and supporting materials in both hard copy and electronically to the RACF, including on-site staff (e.g. nurses and aged care workers) and visiting healthcare professionals (e.g. medical practitioners and allied health). Materials will be distributed by the on-site aged care pharmacist at baseline as well as on request. Distributed materials will include the Guidelines, a companion guide, medication factsheets on the allocated medication context (antipsychotics, benzodiazepines or antidepressants) and a curated inventory of existing resources. All these materials will also be publicly available via MAGICapp, an online platform for publication of guidelines and evidence summaries [12]. The level 1 resources will be standardised, and no tailoring of these resources will be undertaken by the Project Management Team. The level 1 strategy is consistent with standard implementation of most clinical practice guidelines in Australian RACFs.

#### Level 2: Pharmacist-led QUM education activities

The level 2 strategy will involve provision of QUM services and distribution of the Guidelines and supporting materials. QUM services are Australian government-funded pharmacist-led group education activities [30]. The QUM service used in this trial will be developed to align with the Pharmaceutical Society of Australia Guidelines for QUM services and the Guidelines [30]. The QUM service will involve a face-to-face, online or pre-recorded group education activity delivered by an on-site aged care pharmacist, delivered to RACF registered nurses and enrolled nurses and aged care workers directly employed by the aged care provider organisation. Each QUM session will run for up to 70 min. The pharmacist will deliver a QUM service once per quarter, with a total of four QUM services delivered over the 12 months. The four sessions will cover the Guidelines, initiation, adverse events and monitoring and discontinuation relevant to the RACFs’ allocated medication context (Table 2).

**Table 2:** Overview of QUM service to RACFs

		**Overview**	**Key take-home messages**
**Guideline introduction**		Introduction to the Guidelines, including the companion guide, medication factsheets and curated inventory of existing resources	Understand the background of the Guidelines, terminology used within the Guidelines and scope of the Guidelines
**Education activity**	Initiation	Each education activity will begin with a brief overview on the use of the psychotropic medication in people living with dementia and changed behaviours Attendees will work through a case study in small groups. Attendees will present their case study answers, and the facilitator will support group discussion. The facilitator will end the education activity with a brief conclusion of the key take-home messages Each component of the education activity will include interactive components to promote group discussion	The *initiation* education activity takes staff through the risks and benefits of the psychotropic medication for people living with dementia. It teaches nurses and aged care staff the steps they should take prior to considering initiation of the psychotropic medication
	Adverse events and monitoring		The *adverse events and monitoring* education activity reviews some key adverse events of the psychotropic medication and teaches nurses and aged care staff monitoring requirements for the psychotropic medication
	Discontinuation		The *discontinuation* education activity teaches nurses and aged care staff when discontinuation should be considered for the psychotropic medication and monitoring requirements during the discontinuation process

Abbreviations: *RACF*, residential aged care facility; *QUM*, quality use of medicine

The Project Management Team will provide education resources to assist in leading the QUM service, including facilitator notes, presentation slides and a sample presentation script. The QUM service resources have been piloted with a Queensland-based regional aged care provider organisation, not otherwise participating in the trial, to test the feasibility of the resources. The level 2 resources will be standardised, and no local tailoring of materials will be undertaken by the Project Management Team. The level 2 intervention is an example of current best practice in relation to implementation of clinical practice guidelines in Australian RACFs.

#### Level 3: Knowledge broker service

The level 3 strategy will involve the on-site knowledge broker who will develop and lead local action plans, as well as provision of QUM services and distribution of the Guideline materials. The knowledge brokers will be employed at the aged care provider organisation level and will work across each participating RACF for that organisation. The knowledge broker will collaborate with the aged care provider organisation’s Medication Advisory Committee (MAC; a multidisciplinary group of healthcare professionals, carers and residents who implement and evaluate medication management policies and procedures [31]) to develop and implement RACF-specific local action plans underpinned by the i-PARIHS framework [32]. Local action plans will be data driven and informed by the trial indicators, iteratively reviewed and updated each quarter (Table 3). The knowledge broker will be an ‘intermediary’ to facilitate knowledge translation by acting as a knowledge manager, capacity builder and linkage agent [16, 21].

- Knowledge manager activities may include embedding and application of Guideline knowledge into local contexts, policies and procedures, assessing barriers and enablers to Guideline implementation in the local context and preparing a local action plan.
- Capacity builder activities may include developing staff knowledge and skills to facilitate and enable Guideline-concordant decision-making, coordinating education relevant to Guideline implementation and conducting audits and providing feedback using indicators of guideline concordance to evaluate implementation initiatives.
- Linkage agent activities may include facilitating collaboration between medical practitioners, nurses and aged care workers, residents and their families, being a conduit between the Project Management Team and the RACF employed staff and visiting team members, and communicating with other knowledge brokers in the trial.

**Table 3:** Knowledge broker study activity requirements for level 3 intervention

Time point	Reporting and study activity requirement
Baseline	*Training and orientation*: Knowledge broker will participate in training and RACF orientation
	*Local action plan*: Knowledge broker will develop local action plan for each RACF and present to RACF management team, Medication Advisory Committee and Project Management Team
	*QUM service*: Delivery of first QUM service according to group allocations
	*Intervention*: Delivery of knowledge broker services as per local action plan
3-month time point	*Local action plan*: Knowledge broker will review and update the local action plan for each RACF (including reflection on successes and challenges in previous quarter) and present to RACF management team, Medication Advisory Committee and Project Management Team
	*QUM service*: Delivery of second QUM service according to group allocations
	*Intervention*: Ongoing delivery of knowledge broker services as per local action plan Support and evaluation: Engagement in semi-structured interviews and focus groups
6-month time point	*Local action plan*: Knowledge broker will review and update the local action plan for each RACF (including reflection on successes and challenges in previous quarter) and present to RACF management team, Medication Advisory Committee and Project Management Team
	*QUM service*: Delivery of third QUM service according to group allocations
	*Intervention*: Ongoing delivery of knowledge broker services as per local action plan *Support and evaluation*: Engagement in semi-structured interviews and focus groups
9-month time point	*Local action plan*: Knowledge broker will review and update the local action plan for each RACF (including reflection on successes and challenges in previous quarter) and present to RACF management team, Medication Advisory Committee and Project Management Team
	*QUM service*: Delivery of fourth and final QUM service according to group allocations
	*Intervention*: Ongoing delivery of knowledge broker services as per local action plan *Support and evaluation*: Engagement in semi-structured interviews and focus groups
12-month time point	*Local action plan*: Knowledge broker will reflect on success and challenges for previous quarter
	*Study close-out*: Semi-structured interviews and focus groups with knowledge brokers

Abbreviations: *RACF*, residential aged care facility; *QUM*, quality use of medicine

Ongoing fidelity of the intervention delivered by the knowledge brokers will be monitored using the discussion boards, local action plans and monthly meetings with the Project Management Team. Meetings will mostly be conducted via video-teleconferencing (e.g. Zoom), with face-to-face meetings organised if needed.

### Data collection

Data collection from the RACFs will occur at baseline and quarterly (3, 6, 9 and 12 months; Table 4). The data collected in this trial will include de-identified data extracted from existing routinely collected data sources within each RACF. Existing data sources will include National Aged Care Mandatory Quality Indicator Program records [33], psychotropic registers [34], prescribing and medication administration records, nursing progress notes, medical records and behaviour support plans. Data will be collected by trained aged care nurses, health or care professionals, employed or contracted by each of the participating aged care provider organisations, who will be blinded to the RACF group allocations. One or more nurses, health or care professionals per participating aged care provider organisation will attend an online training webinar on data collection led by the Project Management Team. Data will be collected and stored on a secure server by Monash University using REDCap™ (Research Electronic Data Capture), a secure web-based software platform designed to support data capture for research studies [35, 36]. Trial data will be stored for a minimum of 15 years after publication of result in accordance with Australian NHMRC and Monash University guidance for medical research involving clinical trials. The final trial dataset will only be accessible to the trial investigators and statistician or epidemiologist.

**Table 4:** SPIRIT diagram — schedule of enrolment, intervention, and assessment for the EMBRACE trial

Trial period								Data source
**Time point**	Enrolment	Allocation	Intervention period					
	*-t*	*-t*	0	3	6	9	12	
**RACF enrolment**								
Eligibility screen	x							
Consent	x							
Allocation		x						
**Intervention**								
KB recruitment		x						
Intervention implementation			x	x	x	x	x	
**Data collection/assessments**								
RACF-level demographic information			x	x	x	x	x	Clinical and administration records; psychotropic register
RACF-level Guideline concordance			x	x	x	x	x	Prescribing and administration records, nursing progress notes, medical records, psychotropic register, behaviour support plans
RACF-level prevalence of medication and behavioural incidents			x	x	x	x	x	RACF Serious Incident Response Scheme
RACF-level proportions of hospitalisations, falls and major injury, polypharmacy, QoL, ADL			x	x	x	x	x	National Aged Care Mandatory Quality Indicator Program records
KB demographics			x					Knowledge brokers
KB activities			x	x	x	x	x	Local action plans, discussion board, quarterly interviews

Abbreviations: *ADL* activities of daily living, *KB* knowledge broker, *RACF* residential aged care facility, *QoL* quality of life, *-t* prior to baseline

#### RACF-level demographic information

Aggregated RACF-level demographic data will be collected from each RACF using a standard data collection form and will include the following: resident bed-days per quarter, proportion of residents who are female, median/mean resident age, proportion of residents with documented diagnosis of cognitive impairment or dementia and proportion of residents using psychotropic medications (antipsychotics, antidepressants, benzodiazepines, z-drugs, opioids and anticonvulsants).

#### RACF Guideline concordance

Trained nurses and health or care professionals will assess Guideline concordance using a standard data collection form completed online (preferred) or in a paper-based version. The data collection form will include questions to assess concordance with indicators for each medication context (Tables 5, 6 and 7). This will require trained nurses and health or care professionals at each participating aged care provider organisation to assign each resident a unique trial identification number. Each aged care provider organisation will securely store the spreadsheet of each resident’s trial identification number, and this will only be accessed by appropriate staff employed by the aged care provider organisation, as needed for care delivery. The Project Management Team will not have access to this information and will not be able to identify residents.

**Table 5:** Indicators to assess aggregate RACF-level concordance: antipsychotics

	***Indicators to assess aggregate RACF-level concordance***	***Corresponding Guideline recommendations***
1	Residents using antipsychotics have documentation of experiencing distressing psychotic symptoms and/or aggression/agitation within the past 12 weeks that represents a direct threat to either the resident or other residents, staff, or family for the resident’s prescription of the antipsychotic	GPS 9; CR 3
2	Residents using antipsychotics have documentation of individually targeted non-pharmacological management strategies to manage distressing psychotic symptoms and/or aggression/agitation that are currently in place	GPS 6
3	Residents using antipsychotics are using a second-generation antipsychotic	CR 1
4	Residents using antipsychotics have documentation of informed consent for use of the antipsychotic	GPS 8
5	Residents using antipsychotics have a documented date of next review for antipsychotic treatment effectiveness	GPS 10
6	Residents using antipsychotics have a current antipsychotic adverse event monitoring protocol in place	GPS 20
7	Residents using antipsychotics have used the current antipsychotic for less than 12 weeks	GPS 19; GPS 21
8	Residents using antipsychotics for more than 12 weeks are currently having the antipsychotic dose tapered	GPS 19; GPS 23
9	Residents using antipsychotics for more than 12 weeks and not currently tapering have documentation that the resident has been reviewed by either a psychiatrist or geriatrician, a formal documented discussion between the prescriber and a psychiatrist or geriatrician in which both practitioners agree or a documented clinical review involving the prescriber and at least one other medical practitioner in which both practitioners agree	GPS 19; GPS 21; GPS 22

Abbreviations: *RACF*, residential aged care facility; *GPS*, good practice statement; *CR*, conditional recommendation. GPS and CR refer to those in the Clinical Practice Guidelines for the Appropriate Use of Psychotropic Medications in People Living with Dementia and in Residential Aged Care

**Table 6:** Indicators to assess aggregate RACF-level concordance: benzodiazepines for sleep disturbance

	***Indicators to assess aggregate RACF-level concordance***	***Corresponding Guideline recommendations***
1	Residents using benzodiazepines have documentation of individually targeted non-pharmacological management strategies for sleep disturbance that were trialled for an adequate period of time prior to commencing the benzodiazepine	GPS 26
2	Residents using benzodiazepines have documentation of individually targeted non-pharmacological management strategies to manage sleep disturbance that are currently in place	GPS 6
3	Residents using benzodiazepines have documentation of informed consent for use of the benzodiazepine	GPS 8
4	Residents using benzodiazepines have a documented date of next review for benzodiazepine treatment effectiveness	GPS 10
5	Residents using benzodiazepines have a current benzodiazepine adverse event monitoring protocol in place	GPS 28
6	Residents using benzodiazepines have used the current benzodiazepine for less than 2 weeks	GPS 26
7	Residents using benzodiazepines for more than 2 weeks are currently having the benzodiazepine dose tapered	GPS 29
8	Residents using benzodiazepines for more than 2 weeks and not currently tapering have documentation of a review of the harms and benefits of benzodiazepine continuation	CR 6

*Abbreviations*: *RACF*, residential aged care facility; *GPS*, good practice statement; *CR*, conditional recommendation. GPS and CR refer to those in the Clinical Practice Guidelines for the Appropriate Use of Psychotropic Medications in People Living with Dementia and in Residential Aged Care

**Table 7:** Indicators to assess aggregate RACF-level concordance: antidepressants

	***Indicators to assess aggregate RACF-level concordance***	***Corresponding Guideline recommendations***
1	Residents using antidepressants have documentation of moderate or severe major depressive disorder	CR 9
2	Residents using antidepressants have documentation of individually targeted non-pharmacological management strategies that are currently in place	GPS 6
3	Residents using antidepressants have documentation of informed consent for use of the antidepressant	GPS 8
4	Residents using antidepressants have a documented date of next review for antidepressant treatment effectiveness	GPS 10
5	Residents using antidepressants have a current antidepressant adverse event monitoring protocol in place	GPS 36
6	Residents using antidepressants have used the current antidepressant for less than 6 months	CR 11
7	Residents using antidepressants for more than 6 months are currently having the antidepressant dose tapered	GPS 37
8	Residents using antidepressants for more than 6 months and not currently tapering have documentation of a review of the harms and benefits of antidepressants continuation	CR 11

*Abbreviations*: *RACF*, residential aged care facility; *GPS*, good practice statement; *CR*, conditional recommendation. GPS and CR refer to those in the Clinical Practice Guidelines for the Appropriate Use of Psychotropic Medications in People Living with Dementia and in Residential Aged Care

Non-identifiable data will be aggregated by Monash University and transferred to Aspect Health using a secure transfer system for the purpose of computing RACF-level Guideline concordance. These data will be fed-back to participating RACFs on a quarterly basis using an electronic dashboard system developed by Aspect Health. Aspect Health is a Queensland-based medication management provider. Aggregated non-identifiable data will be treated confidentially and stored on Aspect Health’s secure server. Each RACF will be able to view their Guideline concordance, compared against the Guideline concordance across their organisation and compared against national Guideline concordance (i.e. aggregate concordance of all participating RACFs) via a secure, password-protected login process. Aggregated data will be reported as proportions or percentages to avoid any potential for RACF re-identification. Aggregated Guideline concordance will be used by the knowledge broker to evaluate quality improvement initiatives and facilitate benchmarking across RACFs and aged care provider organisations.

#### RACF-level prevalence of psychotropic medication use

RACF-level prevalence of psychotropic medications (including antipsychotics, benzodiazepines, antidepressants, z-drugs, opioids and anticonvulsants) will be calculated by number of residents administered psychotropic medications in the last 7 days (regular and pro re nata (PRN)) divided by total number of residents assessed at the RACF. These data will be extracted from audit reports of the medication records of each RACF.

#### RACF-level proportions of hospitalisation, falls, falls with major injury, polypharmacy, quality of life and activities of daily living

Data on RACF-level proportions of hospitalisation, falls, falls with major injury, polypharmacy, quality of life and activities of daily living will be extracted from the mandatory National Aged Care Quality Indicator Program records into a standard data collection form [37]. The following definitions will be used in line with the National Aged Care Quality Indicator Program:

- Hospitalisation: RACF-level proportion of residents with hospitalisation compared to baseline. Hospitalisation will be assessed as residents who had one or more emergency department presentations or hospital admissions within the last 3 months.
- Falls: RACF-level proportion of residents falls compared to baseline. A fall event (one or more) will be assessed as an event that results in a person coming to rest inadvertently on the ground or other lower level within the last 3 months [37].
- Falls with major injury: RACF-level proportion of residents falls with major injury compared to baseline. Falls with major injury will be assessed as a fall event (one or more) that resulted in a major injury or injuries within the last 3 months.
- Polypharmacy: RACF-level proportion of residents’ polypharmacy compared to baseline. Polypharmacy will be assessed as residents who were prescribed nine or more medications at the time of data collection. Topical preparations, dietary supplements, short-term medications (e.g. short-course antibiotics) and PRN medications will be excluded.
- Quality of life: RACF-level proportion of residents who report “good” or “excellent” quality of life as assessed using the Quality of Life Aged Care Consumers (QOL-ACC) tool compared to baseline [38]
- Activities of daily living: RACF-level proportion of residents who experience a decline in their activities of daily living assessment total score of 1 or more points within the last 3 months. Activities of daily living will be measured using the Barthel Index of Activities of Daily Living [39].

#### RACF-level prevalence of medication and behavioural incidents

Data on RACF-level prevalence of medication incidents and behavioural incidents will be collected from each RACF’s electronic risk management system and Serious Incident Response Scheme (SIRS). As in previous research, medication incidents will be categorised according to the severity and type (e.g. an administration error, adverse drug reaction, resident error, pharmacy dispensing error or prescribing error) [40]. Incidents recorded in SIRS include unreasonable use of force (e.g. hitting, pushing, shoving or rough handling a resident), psychological or emotional abuse and inappropriate use of restrictive practice [41].

#### Knowledge broker demographics

Demographic (age, gender) and professional background (education, qualifications, accreditation status and practice experience) data will be collected from the knowledge brokers at the time of enrolment using a standard data collection form completed online (preferred) or a paper-based version.

#### Knowledge broker activities

Data from local action plans and knowledge broker discussion board posts will be collected throughout the trial period. Data from the local action plans will include the following:

- Goals for improvement (informed by the Guideline indicators)
- Type of local activities implemented
- Resources used to develop activities
- Timeline for implementation of activities
- Whether or not the activity was implemented

Quarterly, semi-structured interviews and/or focus groups with knowledge brokers may also be conducted throughout the trial. Data will be de-identified and qualitatively analysed to inform on outcomes relating to local process evaluations and facilitators and barriers to change.

### Outcomes

#### Primary outcome

The primary outcome will be the proportion of residents with Guideline concordant use of antipsychotics, benzodiazepines and antidepressants measured at the RACF-level. This outcome will be measured at the 6-month time point, informed by a similar study conducted in RACFs [42]. This overall Guideline concordance will be measured as a dichotomous outcome (i.e. concordant vs not concordant among those using the reference medication) (Additional file 2). This trial-specific composite outcome has been developed from a series of indicators for each medication contexts and developed by New South Wales Therapeutic Advisory Group.

#### Secondary outcomes

Secondary outcomes will include:

- Proportion of residents with Guideline concordant use of antipsychotics, benzodiazepines and antidepressants measured at the RACF-level at 3-, 9- and 12-month time points. The 9- and 12-month time points will be used to evaluate intervention sustainability and will be useful for assessing policy and practice change that did not occur at the 6-month time point.
- Proportion of residents concordant with each individual indicator of the Guideline concordance composite outcome for antipsychotics, benzodiazepines and antidepressants use, measured at the RACF level at 3-, 6-, 9- and 12-month time points (Tables 5, 6 and 7).
- Proportion of residents using psychotropic medications (antipsychotics, antidepressants, benzodiazepines, z-drugs, opioids and anticonvulsants) measured at the RACF level at 3-, 6-, 9- and 12-month time points
- Proportion of residents with hospitalisation, falls, falls with major injury, polypharmacy, quality of life and activities of daily living measured at the RACF level at 3-, 6-, 9- and 12-month time points
- Proportion of residents with medication incidents and behavioural incidents measured at the RACF level at 3-, 6-, 9- and 12-month time points

These proportions will be calculated using the total number of residents at each RACF at each of the time points as a denominator.

### Sample size and power

The sample size of 19 RACFs was deemed to have sufficient statistical power. To estimate the statistical power of the helix, counterbalanced, three-strategy, and three-context area (medication type) trial design, we performed 1000 Monte Carlo simulations using Stata/SE Statistical Software: Release 17.0 (StataCorp. 2021). The sample size analysis was conducted at the RACF level based on the primary outcome (i.e. proportion of RACF-level Guideline concordant use of antipsychotics, benzodiazepines and antidepressants). The sample size was based on conducting analysis using a linear mixed model employing fixed, categorical factors for medication type and guideline implementation strategy and RACF as a random intercept. We assumed a minimum important effect size of an absolute change in the proportion of concordant medication category takers of 5% for the level 2 strategy relative to level 1 strategy and of 15% for the level 3 strategy relative to level 1 strategy. Given these assumptions, our sample size of 19 RACFs provides > 99% power to detect the absolute change in proportion of concordant medication category takers of 15% and 81% power to detect the absolute change in proportion of 5%.

### Analysis plan

#### Effectiveness analysis

All primary analyses will be conducted using the intention-to-treat (ITT) principle. Level 3 intervention will be compared to level 1 and level 2 at the 6-month time point as a primary outcome (Table 4).

Per protocol analysis will also be undertaken with the per protocol set including all RACFs without a major protocol deviation. Major protocol deviations may include, but are not limited to, the following:

- Loss of knowledge broker
- Incomplete delivery of intervention (e.g. local action plans, QUM service)

We anticipate that the primary analysis will be conducted using a linear mixed model treating the RACF as a random effect, with the strategy level and medication contexts as fixed effects. Multiple imputation will be used to impute missing covariate values where necessary. Data will be analysed using Stata/SE Statistical Software: Release 17.0 (StataCorp. 2021).

#### Health economic analysis

Health economic analysis will be performed from a societal perspective incorporating costs for hospital transfers, costs of intervention delivery, costs of psychotropic medication use and cost of staff time managing other adverse events (e.g. falls, behaviour-related events). Economic efficiency will be expressed in terms of incremental cost per additional resident who becomes Guideline concordant for a medication context. Costs will be calculated at 2024 value levels. Bootstrap analyses will be conducted to construct 95% confidence ellipses on a cost-effectiveness plane and to undertake acceptability curve analyses.

#### Process evaluation

A mixed method process evaluation will be undertaken using the eight-domain framework published by Grant et al. [43]. Process evaluation will be performed through an analysis of qualitative data (e.g. local action plans, discussion board posts) and quantitative measures of engagement with the intervention (e.g. knowledge broker participation in Project Management Team meetings, frequency of log in events on the Guideline concordance dashboard).

### Dissemination

Findings will be disseminated through lay summaries, conference presentations and peer-reviewed publications. The lay summaries will be co-designed with consumer and end users. All participating aged care provider organisations and RACFs will be provided a final report summarising key findings. Research findings will also be disseminated through member organisations of the EMBRACE stakeholder group and their professional networks.

## Discussion

The EMBRACE trial investigates a novel guideline implementation strategy to improve the implementation of evidence into practice in the RACF setting and directly addresses national and international priority areas: ‘Medication Without Harm’ is the World Health Organization’s (WHO) Third Global Patient Safety Challenge [15]. The EMBRACE trial also addresses dementia as an Australian National Health Priority Area, consistent with the WHO Global Action Plan on Dementia 2017–2025 [44, 45].

Complaints about medications are among the leading sources of complaints to Australia’s Aged Care Quality and Safety Commission [46]. In addressing medication appropriateness, the trial addresses a key concern of consumers and carers. Psychotropic medications contribute to 2.1% of all hospitalisations and 11.3% of all medication harm-related hospitalisations [47]. The trial directly addresses the need for new models of guideline implementation to promote safe and effective psychotropic medication use. Outcomes relating to intervention sustainability are consistent with the need for evidence to inform policy and practice change.

This trial will be the first to evaluate the novel role of system-level knowledge broker pharmacists to facilitate guideline implementation in RACFs. On-site aged care pharmacists are potentially well placed to deliver this intervention, with a recent pharmacist-led multicomponent intervention demonstrating 44% reduction in antipsychotic medication use compared to the control group at 6 months [48]. The knowledge brokers will be on-site and embedded as part of the RACF team [49] and have greater opportunity to lead system-level quality improvement initiatives [21]. The findings of this trial will address the absence of high-quality evidence regarding the effectiveness and cost-effectiveness of knowledge brokers [16] and inform RACF and government funding and resource allocation decisions for potential future roll-out or expansion of the knowledge broker pharmacist role [50].

The EMBRACE trial uses a helix-counterbalanced study design, which provides several advantages over cluster randomised or stepped-wedge designs [19]. Firstly, it is not necessary to adjust for RACF characteristics because each RACF provides data for each implementation strategy. Secondly, all participating RACFs will receive all implementation strategies. This will help to maintain engagement of those RACFs which would otherwise have been randomised to the control group. Finally, the implementation strategies can be delivered simultaneously rather than sequentially as in a stepped-wedge design.

The EMBRACE trial will generate important knowledge about guideline implementation and knowledge brokers that can be used to scale up the intervention across Australia, inform application to other therapeutic areas and contexts, and improve the delivery of healthcare to all residents living in RACFs.

### Supplementary Information

**Additional file 1.** Standard Protocol Items: Recommendations for Interventional Trials (SPIRIT) Checklist.

**Additional file 2.** Concordance with Guideline tool – antipsychotics, benzodiazepines and antidepressants.

## Data Availability

Data sharing is not applicable to this article as no datasets were generated or analysed during the current trial.

## Abbreviations

ANZCTR: Australian New Zealand Clinical Trials Registry
EMBRACE: Evidence-based Medication knowledge Brokers in Residential Aged CarE
i-PARIHS: Integrated-Promoting Action on Research Implementation in Health Services
NHMRC: National Health and Medical Research Council
QUM: Quality use of medicines
RACF: Residential aged care facilities
SIRS: Serious Incident Response Scheme
