# Supplementary material for: Evidence-based Medication knowledge Brokers in Residential Aged CarE (EMBRACE): protocol for a helix-counterbalanced randomised controlled trial
Source: Implement Sci. 2024 Mar 4;19:24. doi: 10.1186/s13012-024-01353-z (PMC10913248; doi:10.1186/s13012-024-01353-z)

Additional file 2.

I. Concordance with Guideline tool: Antipsychotics

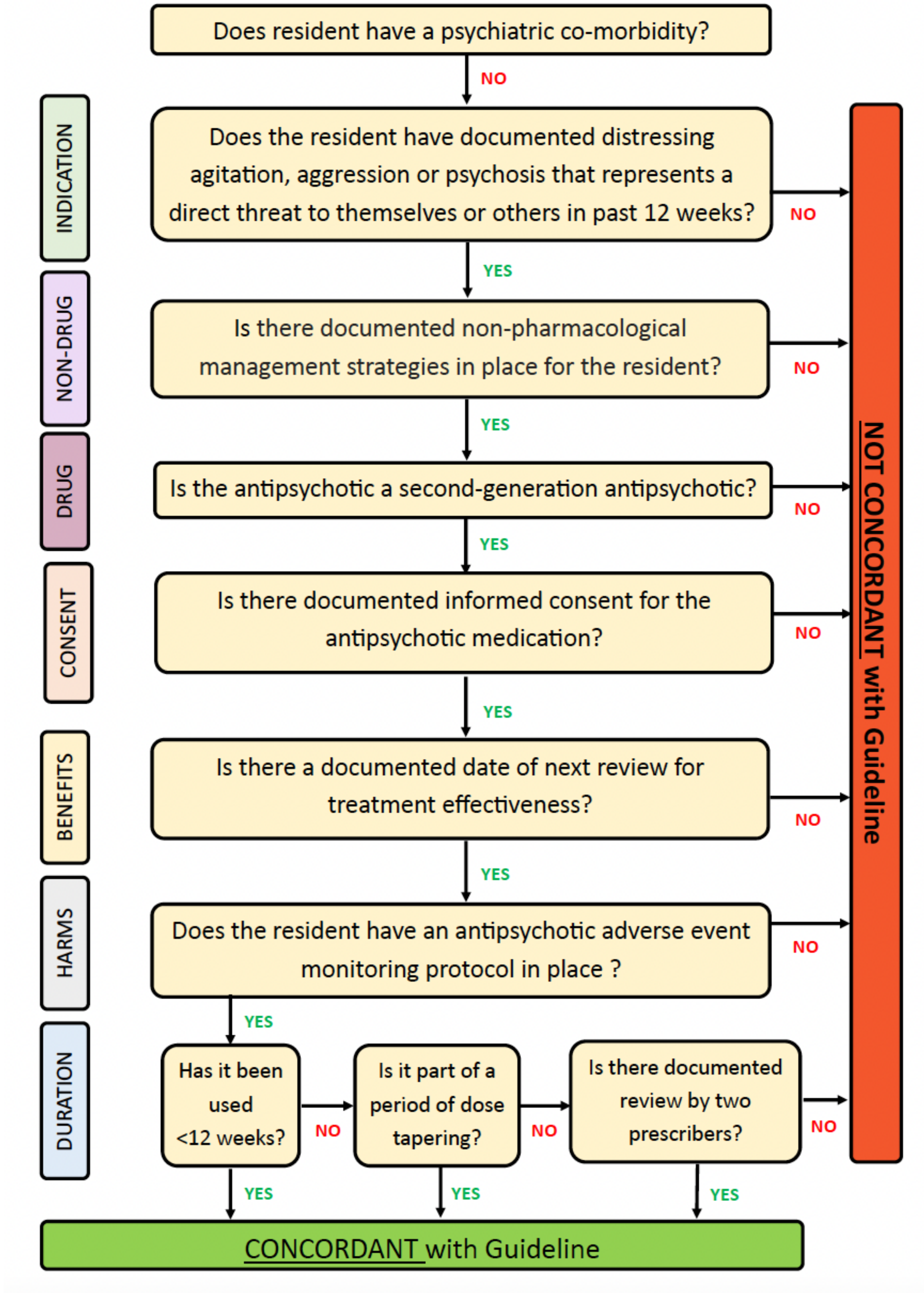

II. Concordance with Guideline tool: Benzodiazepines for sleep

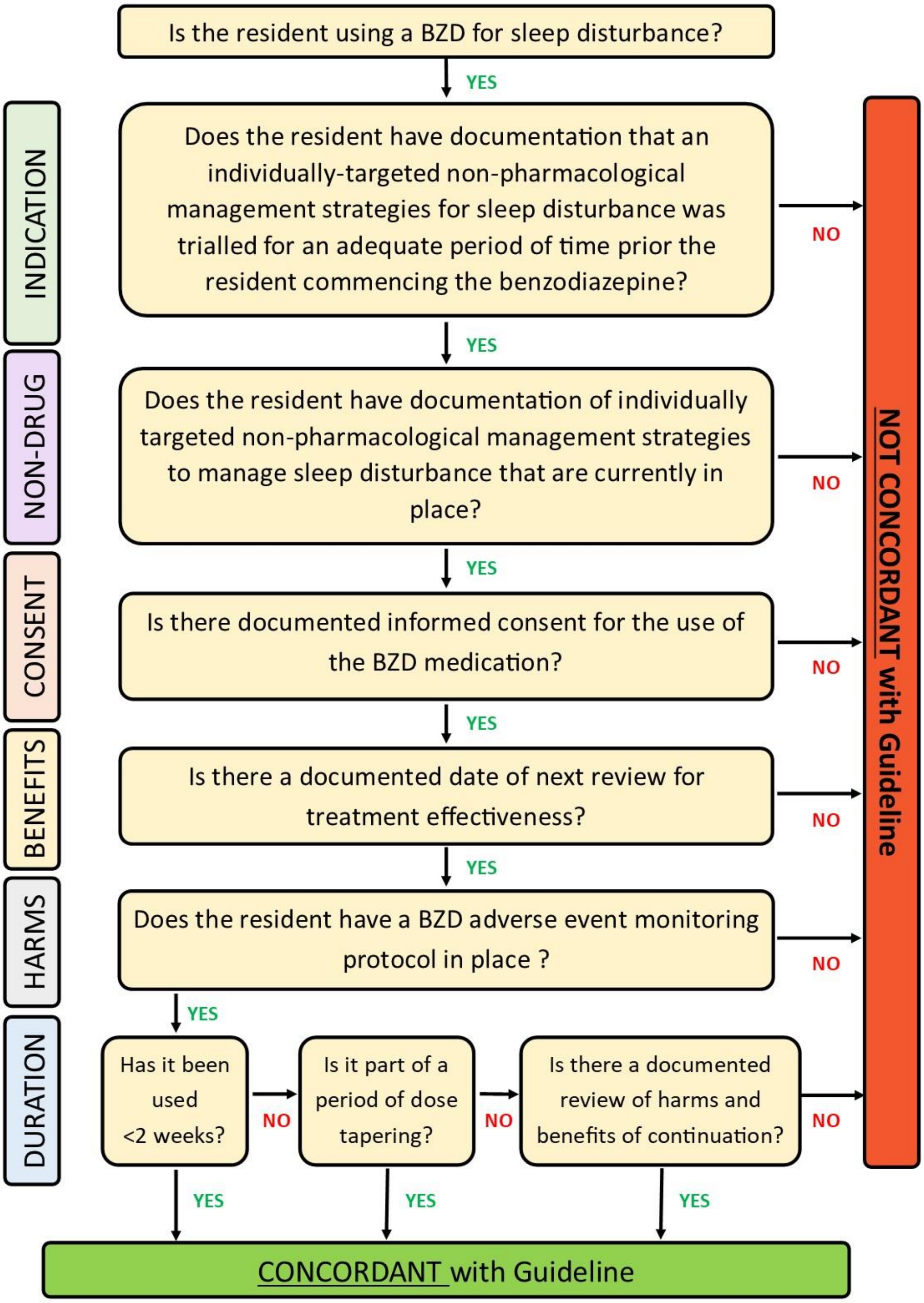

Abbreviations: BZD, benzodiazepines.

III. Concordance with Guideline tool: Antidepressants

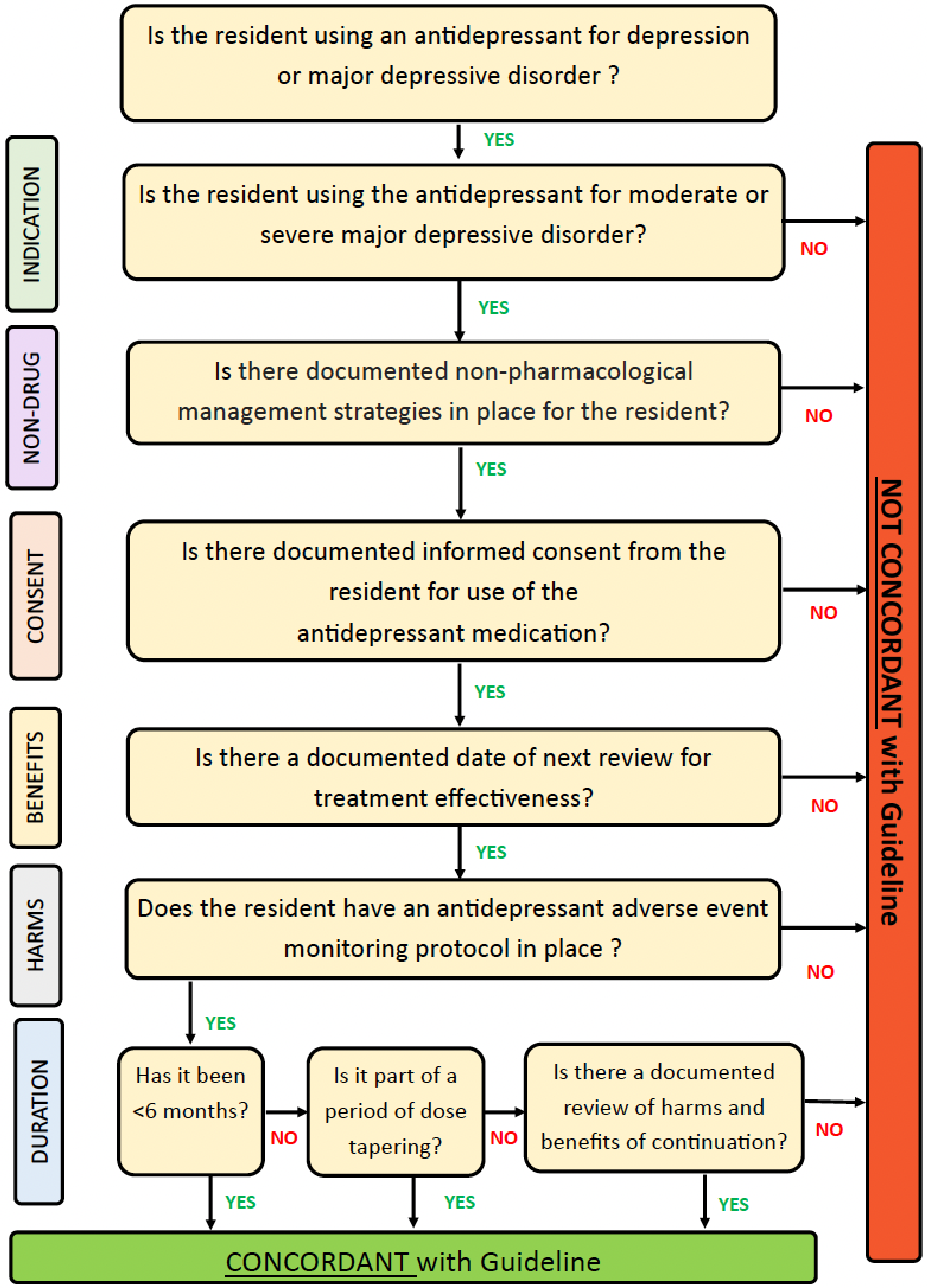

Supplement: Supplementary file 2 — Additional file 2. Concordance with Guideline tool – antipsychotics, benzodiazepines and antidepressants. [file 13012_2024_1353_MOESM2_ESM.pdf]
